# Sex‑specific effects of aging and glycemic control on the association of possible sarcopenia in older adults with type 2 diabetes: a cross-sectional study

**DOI:** 10.1007/s40520-025-03159-5

**Published:** 2025-08-20

**Authors:** Yi-Fang Huang, Shih-Ping Liu, Chih-Hsin Muo, Chen-Yi Lai, Chung-Ta Chang

**Affiliations:** 1 Department of General Dentistry, Chang Gung Memorial Hospital, Linkou, 33305 Taiwan; 2 Graduate Institute of Dental and Craniofacial Science, College of Medicine, Chang Gung University, Taoyuan, 33302 Taiwan; 3 School of Dentistry, College of Oral Medicine, Taipei Medical University, Taipei, 11031 Taiwan; 4 Translational Medicine Research Center, China Medical University Hospital, Taichung, 411 Taiwan; 5 Program for Aging, College of Medicine, China Medical University, Taichung, 404 Taiwan; 6 Management Office for Health Data, China Medical University Hospital, Taichung, 40402 Taiwan; 7 Health Management Center, Far Eastern Memorial Hospital, Taipei, 22056 Taiwan; 8 Graduate Institute of Medicine, Yuan Ze University, Taoyuan, 32003 Taiwan; 9 Department of Emergency Medicine, Far Eastern Memorial Hospital, No.21, Sec. 2, Nanya S. Rd., Banciao Dist., New Taipei, 22056 Taiwan

**Keywords:** Sex difference, Muscle strength, Glycemic control, Dynapenia, AWGS 2019

## Abstract

**Aim:**

This study aimed to investigate sex-specific associations and the synergistic effects of aging and glycemic control on the correlation of possible sarcopenia in older adults with type 2 diabetes mellitus (T2DM).

**Methods:**

In the cross-sectional study of community-dwelling adults aged ≥ 60 years in New Taipei City, Taiwan, Sarcopenia status was classified using the Asian Working Group for Sarcopenia 2019 criteria into possible sarcopenia, sarcopenia, and severe sarcopenia. Categorical and continuous variables were compared using chi-square/Fisher’s exact tests and ANOVA. Sex-specific associations between glycemic control and sarcopenia were examined using logistic regression to estimate odds ratios (ORs) and 95% confidence intervals (CIs).

**Results:**

5,728 participants (mean age 72.1 ± 6.1 years; 47.9% men) were enrolled. After adjustment, older women with T2DM had a higher odds of possible sarcopenia (OR = 2.07, 95% CI = 1.57–2.72). In women aged ≥ 70 years, both good and poor glycemic control were significantly correlated with possible sarcopenia (OR = 1.87 and 2.10; 95% CI = 1.16–3.04 and 1.43–3.07; p for trend < 0.0001). T2DM patients with hypertension, ischemic heart disease (IHD), or depression showed increased sarcopenia association (OR = 1.71, 1.81, and 2.45, respectively). Poor glycemic control revealed increased odds of sarcopenia in those with hypertension (OR = 1.80) or IHD (OR = 2.42).

**Conclusions:**

Glycemic control could be crucial in preventing possible sarcopenia, especially among older women with T2DM. Comorbid hypertension, IHD, or depression are significantly associated with sarcopenia in T2DM patients, particularly in the presence of poor glycemic control.

## Introduction

Sarcopenia is an age-related disease featured by generalized decreased skeletal muscle mass, and loss of muscle strength and/or muscle performance [1]. It has been proved to be associated with increased risk of falls and fractures [1], hospitalization [2], poor quality of life, disability [3], and mortality [4]. Possible sarcopenia, the reduction of skeletal muscle strength with or without decreased muscle performance, is a premorbid status between non-sarcopenia and sarcopenia [1, 5]. Loss of skeletal muscle strength could increase the risk of all-cause mortality, cardiovascular mortality, myocardial infarction, strokes, and injuries from falls [6]. The critical role of possible sarcopenia was emphasized, and early intervention to prevent the deterioration of this entity was recommended in the latest consensuses of both the Asian Working Group for Sarcopenia (AWGS) and the European Working Group on Sarcopenia in Older People (EWGSOP) [1, 5]. Since the rising prevalence of possible sarcopenia and sarcopenia is expected as the world population ages, the bidirectional transitional nature of sarcopenia status [7], risk factor identification, early diagnosis, and aggressive treatment for possible sarcopenia are imperative for health promotion in the older adults.

Diabetes mellitus (DM) presents a high prevalence in older adults, and it is a major contributor to many disorders, such as strokes, Parkinson’s disease, liver disease, chronic kidney disease (CKD), and malignancies [8–11]. Although a relatively high prevalence of sarcopenia in patients with DM has been documented [12–14] and the importance of glucose control for sarcopenia prevention indicated in an animal experiment [15], owing to the smaller sample size in previous research [16–19] several clinically important issues, such as the impact of sex-specific effect and glycemic control status on the development of possible sarcopenia and sarcopenia in DM patients, are inconclusive. Some researchers reported a remarkably high prevalence of sarcopenia in females [17] or males [18], but other researchers indicated no sex distribution difference for sarcopenia among patients with diabetes [19–21]. Sugimoto et al. stated that the glycosylated hemoglobin (HbA1c) level was correlated with a low skeletal mass index (SMI) rather than weak muscle strength or slow gait speed [19], but in another study, Izzo and coworkers concluded no relation between the HbA1c level and sarcopenia in patients with diabetes [22]. Although recent evidence suggested DM, hypertension, heart disease, osteoporosis, and depression could be associated with sarcopenia development [13, 23, 24], studies assessing whether older adults with diabetes who concurrently have these comorbidities are at increased risk of possible sarcopenia and sarcopenia remain very limited, especially given the rising prevalence of multimorbidity among older diabetic patients.

This study primarily aimed to assess whether the association of possible sarcopenia and sarcopenia differs among older adults with type 2 diabetes mellitus (T2DM) according to sex, age group, and glycemic control status, and to investigate whether these factors have a synergistic effect on the statuses of possible sarcopenia and sarcopenia. Additionally, the relationship between common comorbidities—including hypertension, ischemic heart disease (IHD), depression, osteoporosis, and CKD—and the odds of sarcopenia across different glycemic control statuses in individuals with T2DM were assessed.

## Methods

### Participants

This retrospective, cross-sectional observational study was conducted in accordance with the ethical principles outlined in the 1964 Declaration of Helsinki and approved by the Ethics Committee of Far Eastern Memorial Hospital, New Taipei City, Taiwan (Approval No. 112143-F). Data were collected from community-dwelling older adults aged ≥ 60 years who participated in a health screening program in New Taipei City between January 1, 2015, and December 31, 2022.

Participants with type 1 diabetes mellitus (DM), Parkinson’s disease, dementia, stroke, severe cognitive impairment, disability, cachexia, or incomplete data were excluded. A total of 5,728 eligible individuals were included in the analysis. All participants had undergone standardized health assessments conducted by trained medical personnel as part of the screening program. As this study involved the secondary use of de-identified health data, individual informed consent was waived by the ethics committee.

### Assessment

#### Assessment of sarcopenia

Sarcopenia was assessed by handgrip strength, SMI, and physical performance based on the measurement guidelines of the AWGS [25]. Handgrip strength was measured in kilograms (kg). A Smedley dynamometer (Takei Ltd., Niigata, Japan) was used on the dominant hand with each subject’s elbow fully extended. Three trials were conducted, and the highest value was recorded for analysis. The value of handgrip less than 18.0 kg for women and 28.0 kg for men were classified as low muscle strength [1]. A multifrequency bioelectrical impedance analyzer (BC-418; Tanita, Tokyo, Japan) was applied to measure the appendicular skeletal muscle mass (ASM). The SMI was assessed by dividing the calculated ASM by the square of the body height (m^2^). An SMI less than 5.7 kg/m^2^ for women and 7.0 kg/m^2^ for men was classified as low muscle mass [1]. A 6-metre walk was executed to evaluate physical performance. The participant was requested to walk at a normal pace for 6 meters. A walking speed less than 1 m/s was classified as a slow gait speed [1]. According to the consensus of AWGS 2019 [1], these older adults were categorized into four mutually exclusive groups—“normal,” “possible sarcopenia,” “sarcopenia,” and “severe sarcopenia.” “Normal” was defined as the absence of low muscle strength, low muscle mass, and slow gait speed. “Overall sarcopenia” was defined as the presence of any non-normal category.

#### Glycemic control status

The HbA1c level was measured after obtaining the blood sample from patients with T2DM by veinous puncture. The T2DM patients with HbA1c values less than 7% were classified as good control. The T2DM patients with HbA1c values 7% or above were classified as poor control [26].

#### Comorbidities, and other variables

The medical histories of these older adults, including diabetes, hypertension, IHD, depression, and osteoporosis, were recorded through face-to-face interviews with well-trained investigators. An additional double-check was accomplished through medical records to verify the accuracy as much as possible.

The subjects with CKD were diagnosed based on the guidelines of Kidney Disease Improving Global Outcomes (KDIGO) [27]. The period of kidney structure or function abnormalities for the confirmation of CKD was acquired by the medical record review and self-report of the subject. We obtained the blood samples via veinous puncture. The estimated glomerular filtration rate (eGFR) was calculated using the Berlin Initiative Study (BIS) equation after serum creatinine was gauged [28]. An eGFR value less than 60 ml/min/1.73 m^2^ was classified as CKD [29].

Demographic data, such as sex and age, were acquired at the study entry. These older participants were stratified into two age groups (60–69 years old and **≥** 70 years old). Anthropometric parameters, including body weight and body height, were gauged by well-trained investigators.

#### Statistical analyses

Number and percentage were presented for the categorical variables (including sex and comorbidity), and mean and standard deviation (SD) were shown for the continuous variables [including age and body mass index (BMI)]. To use the chi-square test and Fisher’s exact test for testing the difference of categorical variables and analysis of variance (ANOVA) for continuous variables among the three sarcopenia statuses.

Polytomous (multinomial) logistic regression was used to assess the sex-specific association between the three sarcopenia statuses and glycemic control status after adjustment for age, hypertension, IHD, depression, osteoporosis, CKD, and BMI. Logistic regression was used to estimate the sex-specific association between overall sarcopenia and the glycemic control status after adjustment for age, hypertension, IHD, depression, osteoporosis, CKD, and BMI. We assessed the joint effect of overall sarcopenia between T2DM with hypertension, IHD, depression, osteoporosis, and CKD. Age and sex stratified analyses were also assessed. All statistical analyses were performed using SAS, version 9.4 (SAS Institute, Cary, North Carolina), and the statistical significance was defined as *p*-value < 0.05.

## Results

**Fig. 1:**
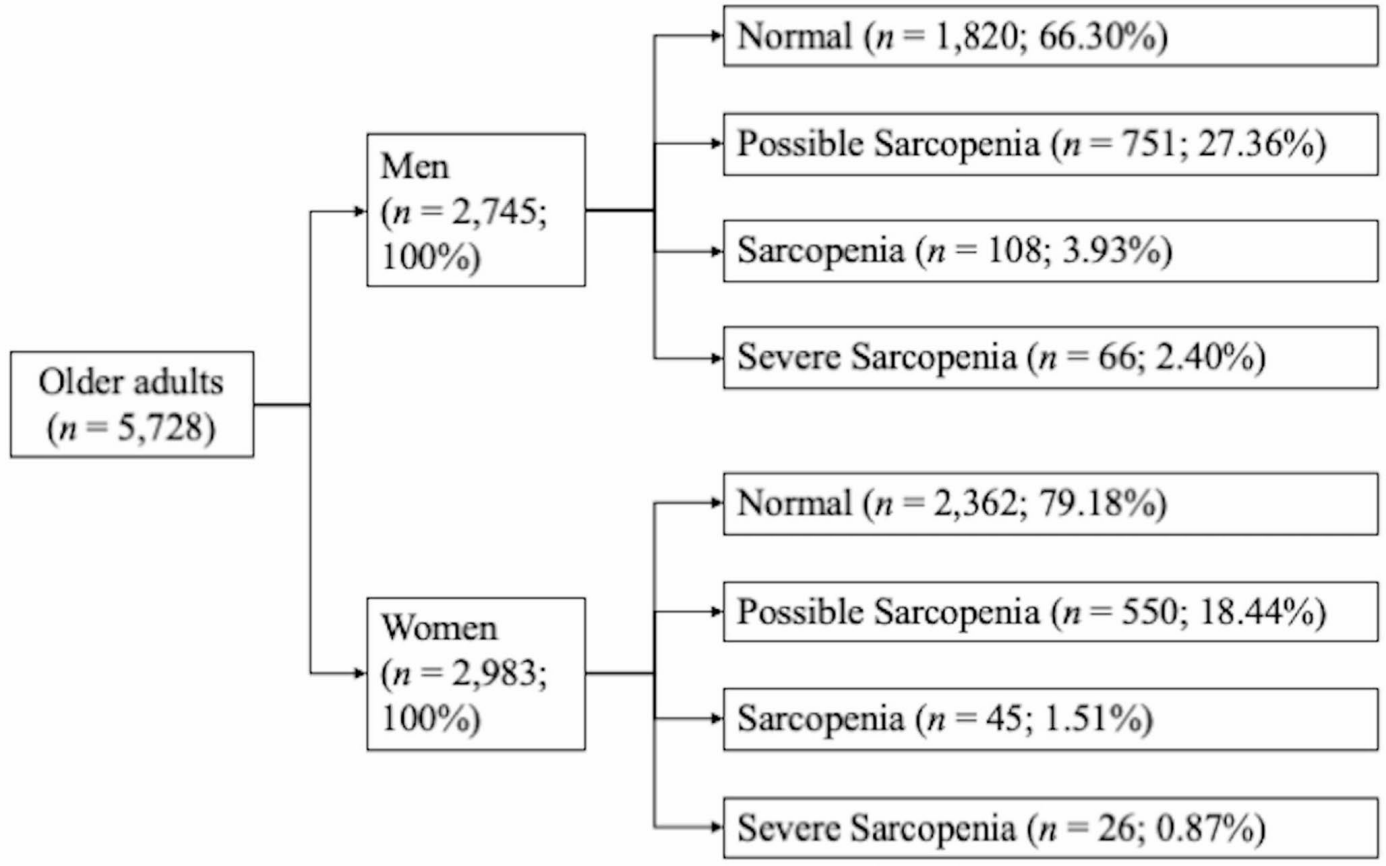
Sex‑specific distribution of sarcopenia status of the study participants

A total of 5,728 older participants was collected in this study (mean age 72.1 ± 6.12 years; men 47.9%): 4,182 were in the normal group, 1,301 were in the possible sarcopenia group, 153 were in the sarcopenia group, and 92 were in the severe sarcopenia group (Table 1). The age increased with the severity of sarcopenia increasing from 70.8 ± 5.08 to 81.2 ± 7.92 years old. Possible sarcopenia patients had the most comorbidity, including T2DM (23.5%), hypertension (7.61%), IHD (4.92%), osteoporosis (2.46%), and CKD (24.8%). However, severe sarcopenia patients had the most depression (5.43%). Sarcopenia patients had the lowest BMI (20.1 ± 2.85). A detailed sex‑specific distribution of sarcopenia statuses is presented in Fig. 1.

**Table 1:** Distribution of demographics in study subjects

Variable	Normal *N* = 4,182		Possible Sarcopenia *N* = 1,301		Sarcopenia *N* = 153		Severe sarcopenia *N* = 92		*p*-value
Age, mean (SD)	70.8	(5.08)	75.2	(7.10)	75.8	(6.49)	81.2	(7.92)	< 0.0001
Men, *n* (%)	1820	(43.5)	751	(57.7)	108	(70.6)	66	(71.7)	< 0.0001
Comorbidity, *n* (%)									
T2DM	681	(16.3)	306	(23.5)	19	(12.4)	11	(12.0)	< 0.0001
Good control	207	(4.95)	99	(7.61)	8	(5.23)	3	(3.26)	< 0.0001
Poor control	474	(11.3)	207	(15.9)	11	(7.19)	8	(8.70)	
Hypertension	1067	(25.5)	431	(33.1)	22	(14.4)	19	(20.7)	< 0.0001
IHD	131	(3.13)	64	(4.92)	4	(2.61)	1	(1.09)	0.010
Depression*	75	(1.79)	36	(2.77)	2	(1.31)	5	(5.43)	0.020
Osteoporosis*	52	(1.24)	32	(2.46)	2	(1.31)	1	(1.09)	0.022
CKD	597	(14.3)	322	(24.8)	26	(17.0)	22	(23.9)	< 0.0001
BMI, mean (SD)	24.3	(2.95)	24.6	(2.88)	20.1	(2.85)	20.5	(2.15)	< 0.0001

1 missing in weight (1 in normal)

Poor control: HbA1c ≥ 7

CKD, eGFR < 60

*Fisher exact test

Table 2 presents the relationship between the three sarcopenia statuses and the glycemic control status, stratified by sex. In the older women group, T2DM patients had a higher correlation of overall sarcopenia compared to non-T2DM patients after adjusting for age, hypertension, IHD, depression, osteoporosis, CKD, and BMI (OR = 2.01, 95% CI = 1.53–2.63), no matter the glycemic control status. Additionally, female participants with T2DM presented a higher odds of possible sarcopenia (OR = 2.07, 95% CI = 1.57–2.72), but this association was not present in the older male group.

The joint effect of overall sarcopenia between glycemic control statuses with sarcopenia-associated risk factors is shown in Table 3. Patients with T2DM and hypertension had the significantly highest overall sarcopenia association when compared to patients without T2DM and hypertension (OR = 1.71, 95% CI = 1.38–2.13). Similar results were presented in the T2DM patients with IHD (OR = 1.81, 95% CI = 1.03–3.17) and the T2DM patients with depression (OR = 2.45, 95% CI = 1.11–5.40) groups.

Regardless of age, women with T2DM had a significantly higher overall sarcopenia association compared to women without T2DM (OR = 2.08 and 1.91, 95% CI = 1.31–3.30 and 1.39–2.61 for < 70 and ≥ 70 years old, respectively) (Table 4). Female T2DM patients of any age had a higher possible sarcopenia association, but there was no significant association between T2DM and sarcopenia for male patients.

**Table 2:** Odds ratio of sarcopenia and glycemic control status by sex

	Possible Sarcopenia		Sarcopenia		Severe sarcopenia		Overall sarcopenia	
Variable	OR (95% CI)	*p*-value	OR (95% CI)	*p*-value	OR (95% CI)	*p*-value	OR (95% CI)	*p*-value
Men								
T2DM	1.17 (0.90–1.53)	0.231	1.53 (0.81–2.89)	0.195	1.36 (0.56–3.30)	0.491	1.20 (0.94–1.55)	0.147
Good control	1.09 (0.71–1.66)	0.697	1.33 (0.51–3.49)	0.563	0.34 (0.04–2.81)	0.316	1.08 (0.72–1.62)	0.711
Poor control	1.21 (0.90–1.63)	0.206	1.63 (0.76–3.47)	0.209	2.15 (0.84–5.51)	0.113	1.26 (0.95–1.68)	0.110
*p* for trend	0.202		0.183		0.211		0.112	
Women								
T2DM	2.07 (1.57–2.72)	< 0.0001	0.61 (0.16–2.37)	0.473	0.91 (0.23–3.65)	0.897	2.01 (1.53–2.63)	< 0.0001
Good control	2.18 (1.45–3.28)	0.0002	1.03 (0.19–5.50)	0.971	1.55 (0.29–8.37)	0.608	2.13 (1.43–3.19)	0.0002
Poor control	2.01 (1.46–2.77)	< 0.0001	0.33 (0.04–2.99)	0.323	0.50 (0.06–4.38)	0.530	1.94 (1.41–2.67)	< 0.0001
*p* for trend	< 0.0001		0.351		0.671		< 0.0001	

Adjusted for age, hypertension, IHD, depression, osteoporosis, CKD, and BMI

Interaction *p* for possible sarcopenia with T2DM and glycemic control status was < 0.05

Overall sarcopenia combined possible sarcopenia, sarcopenia, and severe sarcopenia group

**Table 3:** Joint effect of glycemic control status and frequent comorbidity on the risk of overall sarcopenia in older adults

	T2DM							
	None		Good control		Poor control		Overall	
Variable	OR (95% CI)	*p*-value	OR (95% CI)	*p*-value	OR (95% CI)	*p*-value	OR (95% CI)	*p*-value
Hypertension								
No	1.00		1.79 (1.17–2.73)	0.007	1.49 (1.09–2.03)	0.013	1.58 (1.22–2.05)	0.0005
Yes	1.17 (0.97–1.42)	0.101	1.54 (1.07–2.22)	0.019	1.80 (1.39–2.32)	< 0.0001	1.71 (1.38–2.13)	< 0.0001
IHD								
No	1.00		1.57 (1.17–2.10)	0.003	1.47 (1.18–1.83)	0.0007	1.50 (1.24–1.81)	< 0.0001
Yes	0.98 (0.61–1.57)	0.926	0.80 (0.26–2.51)	0.705	2.42 (1.26–4.65)	0.008	1.81 (1.03–3.17)	0.038
Depression								
No	1.00		1.48 (1.11–1.98)	0.008	1.55 (1.24–1.92)	< 0.0001	1.52 (1.26–1.84)	< 0.0001
Yes	1.78 (1.06-3.00)	0.031	4.98 (0.78–31.7)	0.089	2.07 (0.86–5.03)	0.107	2.45 (1.11–5.40)	0.026
Osteoporosis								
No	1.00		1.48 (1.11–1.98)	0.008	1.54 (1.24–1.90)	< 0.0001	1.52 (1.26–1.83)	< 0.0001
Yes	1.41 (0.83–2.41)	0.206	3.72 (0.70–19.8)	0.123	1.26 (0.21–7.61)	0.803	2.23 (0.67–7.44)	0.206
CKD								
No	1.00		1.57 (1.14–2.18)	0.007	1.59 (1.24–2.03)	0.0002	1.58 (1.28–1.95)	< 0.0001
Yes	0.95 (0.78–1.17)	0.646	1.24 (0.72–2.15)	0.446	1.31 (0.91–1.87)	0.146	1.29 (0.94–1.76)	0.113

Manually adjusted for age, hypertension, IHD, depression, osteoporosis, CKD, and BMI

Overall sarcopenia combined possible sarcopenia, sarcopenia, and severe sarcopenia group

**Table 4:** Odds ratio of sarcopenia and glycemic control statuses stratified by sex and age

	Possible Sarcopenia		Sarcopenia		Severe sarcopenia		Overall sarcopenia	
Variable	OR (95% CI)	*p*-value	OR (95% CI)	*p*-value	OR (95% CI)	*p*-value	OR (95% CI)	*p*-value
Men < 70 years								
T2DM	1.31 (0.87–1.98)	0.200	1.80 (0.61–5.34)	0.287	3.55 (0.55-23.0)	0.185	1.37 (0.93–2.02)	0.112
Good control	1.30 (0.67–2.50)	0.440	0.73 (0.09–6.10)	0.769	NA		1.20 (0.63–2.27)	0.581
Poor control	1.34 (0.83–2.14)	0.230	2.64 (0.81–8.61)	0.108	6.11 (0.89–41.8)	0.065	1.45 (0.94–2.25)	0.096
*p* for trend	0.200		0.145		0.084		0.092	
Men ≥ 70 years								
T2DM	0.90 (0.65–1.24)	0.509	1.22 (0.55–2.70)	0.631	0.67 (0.25–1.76)	0.414	0.88 (0.65–1.20)	0.410
Good control	0.81 (0.48–1.38)	0.436	1.49 (0.49–4.49)	0.483	0.30 (0.04–2.20)	0.226	0.82 (0.50–1.35)	0.441
Poor control	0.90 (0.62–1.30)	0.573	1.03 (0.37–2.87)	0.956	0.84 (0.29–2.41)	0.744	0.90 (0.64–1.29)	0.576
*p* for trend	0.493		0.772		0.526		0.496	
Women < 70 years								
T2DM	2.20 (1.38–3.51)	0.0009	2.48 (0.29–21.5)	0.410	NA		2.08 (1.31–3.30)	0.002
Good control	3.15 (1.63–6.11)	0.0007	7.32 (0.82–65.3)	0.075	NA		3.22 (1.69–6.14)	0.0004
Poor control	1.67 (0.94–2.95)	0.078	NA		NA		1.60 (0.91–2.82)	0.105
*p* for trend	0.016		0.734				0.019	
Women ≥ 70 years								
T2DM	2.02 (1.47–2.79)	< 0.0001	0.32 (0.05–1.91)	0.210	0.71 (0.17–3.06)	0.650	1.91 (1.39–2.61)	< 0.0001
Good control	1.87 (1.16–3.04)	0.011	0.29 (0.02–3.68)	0.342	1.01 (0.16–6.30)	0.993	1.79 (1.12–2.85)	0.016
Poor control	2.10 (1.43–3.07)	0.0001	0.29 (0.03–3.21)	0.311	0.48 (0.05–4.36)	0.517	1.98 (1.36–2.89)	0.0004
*p* for trend	< 0.0001		0.198		0.558		< 0.0001	

Adjusted for HTN, IHD, depression, osteoporosis, CKD, and BMI

Interaction *p* for possible sarcopenia with T2DM and glycemic control status was < 0.05

Overall sarcopenia combined possible sarcopenia, sarcopenia, and severe sarcopenia group

## Discussion

This study demonstrated a sex-specific distribution for the odds of possible sarcopenia rather than sarcopenia in older adults with T2DM. Older women with T2DM had a significantly higher association of possible sarcopenia, and a positive correlation was identified between possible sarcopenia and glycemic control status in older women (age **≥** 70 years) with T2DM. The study found that patients with T2DM who also have hypertension, IHD, or depression are at a significantly increased odds of sarcopenia, suggesting a potential synergistic effect between T2DM and these comorbid conditions.

The role of sex differences in the development of sarcopenia in older adults with DM is controversial. Two meta-analysis studies [16, 24] and one United States survey [30] reported that the male sex is a predictor for sarcopenia in patients with diabetes. One Japanese study conducted by Mori et al. reported that the female sex is a prominent risk factor for possible sarcopenia rather than sarcopenia in the T2DM population [31]. Our result showed a 2.01-fold increased odds for overall sarcopenia in older women with T2DM (Table 2). Since older Asian women often engage in fewer aerobic and resistance exercises than Western women, differences in the lifestyle of the study subjects, the measurement methods of muscle quantity and quality, the definition of sarcopenia, and ethnicity could be the possible reasons for the discord between the aforementioned investigations. In our subgroup analysis, a significant association of possible sarcopenia in the female participants with T2DM was recognized (adjusted OR = 2.07, Table 2). This phenomenon may be due to the pathological change in the micro-environment of circulation and innervation, underlying metabolic dysregulation or comorbidity burden [32], but not HbA1c control itself. Because of the relatively smaller sample size of sarcopenia and severe sarcopenia in our survey (Fig. 1), we are in agreement with the observation of Mori et al. [31], that we could only indicate older female patients with T2DM have a significantly higher odds of possible sarcopenia. The association between sex and sarcopenia could not be concluded. More population-based prospective studies with enough sarcopenia and severe sarcopenia cases to determine the impact of sex differences on the risk of sarcopenia in patients with DM are suggested in the future.

Handgrip strength could be a valuable prognostic predictor for several diseases [31]. Decreased handgrip strength is frequently observed in DM patients [16, 31, 33, 34]. The association of handgrip strength reduction with poor glycemic control may imply skeletal muscle weakness, which is proposed to be an important DM complication in addition to the microvascular complications of diabetes [33, 35]. Divergent definitions, measurement methods, and cut-off values for skeletal muscle weakness prohibited the correct evaluation of the association between skeletal muscle weakness and DM [16, 33, 35]. Possible sarcopenia, which was introduced by AWGS 2019 [1], provided the latest definition, measurement methods, and cut-off values for skeletal muscle weakness, which could fill the gap.

Two recent studies using the algorithm of AWGS 2019 to classify the statuses of sarcopenia found that T2DM is an independent risk factor of possible sarcopenia rather than sarcopenia [31, 34]. Nishimoto et al. further concluded a positive correlation between pre-DM and DM patients on the risk of possible sarcopenia, but a similar correlation could not be observed in their sarcopenia population [34]. However, they did not estimate the impact of sex and glycemic control status on the risk of possible sarcopenia and sarcopenia. In another small sample size study, Mori and coworkers identified age ≥ 65 years old and female sex rather than HbA1c ≥ 8.0% as risk factors of possible sarcopenia in T2DM patients, but the contribution of sex difference dissipated in their sarcopenia risk factor evaluation [31]. Our population presented a similar mean age, sex distribution, and prevalence of sarcopenia to the large sample size study of Nishimoto and colleagues [34]. We are not only in agreement with the results of the above two studies that T2DM is correlated with the risk of possible sarcopenia rather than sarcopenia, but we further found a 2.07-fold increased odds of possible sarcopenia in older T2DM women after adjustment (Table 2), which is supported by Mori et al. [31]. It is noteworthy that the older female participants (aged ≥ 70 years) with poor glycemic control (HbA1c ≥ 7.0%) group showed the greatest odds of possible sarcopenia (adjusted OR = 2.10, *p* < 0.0001), and the odds ratios for possible sarcopenia were significantly positively correlated between the good and poor glycemic control groups in the older female population was first identified (*p* for trend < 0.0001) in the subgroup analysis (Table 4). The discrepancy between our work and the analysis of Mori et al. on the role of glycemic control in the risk of possible sarcopenia may be due to differences in the study sample size and HbA1c cut-off values. Larger sample-size prospective studies to define an optimal glycemic control level for sarcopenia prevention are required in the future.

While many studies have identified DM, osteoporosis, heart disease, and depression as important risk factors for sarcopenia [12, 13, 23], it remains unclear whether the presence of osteoporosis, heart disease, or depression further increases the risk of sarcopenia in patients with DM. In this study, the significant joint effect for the odds of sarcopenia in T2DM patients with hypertension, IHD, or depression was first discovered. We further identified a potential positive correlation between good and poor glycemic control for the association of sarcopenia in older T2DM patients with hypertension or IHD (Table 3). These novel findings may advise clinical practitioners regarding the possibility of synergism of T2DM and other comorbidities on the association for sarcopenia, especially since the association between hypertension and sarcopenia is currently inconclusive [23, 36]. We therefore suggested the importance of glycemic control in patients with T2DM. Proper glycemic control may reduce the impact of hypertension or IHD on the correlation of sarcopenia in T2DM people. A previous work mentioned patients with osteoporosis are at a higher risk for sarcopenia [12]. In this study, owing to the relatively smaller sample size of osteoporosis participants, we could not accurately estimate the impact of osteoporosis on the risk of sarcopenia in the T2DM population. Further large-scale prospective cohort studies to determine the joint effect between DM and osteoporosis are warranted in the future.

Two recent studies indicated the characteristics of bidirectional transitions of sarcopenia status [7], and that adequate nutrition and sufficient aerobic and resistance exercises could improve possible sarcopenia [37], highlighting the importance of risk factor prevention, early identification, and aggressive intervention for sarcopenia in clinical settings. Since the complex underlying interaction mechanism beneath these comorbidities, such as hypertension, IHD, depression, and diabetes, sarcopenia was suggested to be an important predictor for the progression of several diseases [6]. Taken together, the improvement of sarcopenia status via aggressive intervention might be helpful for disease control of older patients with these comorbidities.

In this study, we identified a significant association between women and possible sarcopenia among patients with T2DM. Notably, we were the first to clearly demonstrate the critical role of glycemic control in the association of possible sarcopenia among older diabetic women aged ≥ 70 years. Additionally, in older adults with T2DM who also suffer from hypertension or IHD, poor glycemic control status was found to be significantly associated with an elevated risk of sarcopenia.

This study has several limitations. First, due to its cross-sectional design, causal relationships among sex, glycemic status, aging, and the risk of possible sarcopenia in patients with T2DM cannot be established. Second, although the results showed a significant sex‑specific association with overall sarcopenia in older T2DM women, only the sex‑specific association between possible sarcopenia and older T2DM women could be indicated. The correlation between sarcopenia and older T2DM women could not be defined due to the small sample sizes for sarcopenia and severe sarcopenia, which could limit the statistical power in sex-stratified analyses. Third, the study population may represent a relatively healthier subset of older adults, as participation required the ability to attend community-based screenings without assistance. Individuals with severe comorbidities or substantial physical impairments may have been underrepresented. Nevertheless, the age, sex distribution, and sarcopenia prevalence observed in our cohort are comparable to those reported in the community-based survey by Nishimoto et al. [34], suggesting some degree of generalizability. Despite this, the potential for selection bias inherent in community-based studies cannot be fully excluded. Another limitation of this study is the lack of analysis on the potential risk of sarcopenia associated with antidiabetic medications. In future research, we aim to investigate the possible association between different antidiabetic treatments and the risk of sarcopenia in patients with diabetes. To better elucidate the roles of sex, glycemic control, aging, and comorbidities in the development of sarcopenia among patients with DM, future prospective, population-based studies incorporating both community- and clinical-setting populations are warranted.

## Conclusions

This study highlighted the significant association between glycemic control and possible sarcopenia in diabetic older women, especially in older female DM patients aged ≥ 70 years old with poor glycemic control status. The joint effect of several comorbidities, including hypertension, IHD, and depression, with DM on the association of sarcopenia to prevent further disability and mortality was emphasized. Due to the bidirectional transitional nature of sarcopenia status, early prevention and intervention of possible sarcopenia is suggested.

## Data Availability

No datasets were generated or analysed during the current study.
